# Prevalence and risk factors of kidney stone disease in population aged 40–70 years old in Kharameh cohort study: a cross-sectional population-based study in southern Iran

**DOI:** 10.1186/s12894-022-01161-x

**Published:** 2022-12-19

**Authors:** Leila Moftakhar, Fatemeh Jafari, Masoumeh Ghoddusi Johari, Ramin Rezaeianzadeh, Seyed Vahid Hosseini, Abbas Rezaianzadeh

**Affiliations:** 1 Student Research Committee, Shiraz University of Medical Sciences, Shiraz, Iran; 2 Breast Diseases Research Center, Shiraz University of Medical Sciences, Shiraz, Iran; 3 Experimental Medicine Program, Department of Medicine, Faculty of Medicine, University of British Columbia, Vancouver, BC Canada; 4 Colorectal Research Center, Shiraz University of Medical Sciences, Shiraz, Iran

**Keywords:** Prevalence, Risk factor, Kidney stone, PERSIAN cohort

## Abstract

**Background:**

Kidney stone is the major cause of morbidity, and its prevalence is increasing in the world. This study aimed to assess the prevalence and risk factors of kidney stone in the adult population of southern Iran based on the data of the Kharameh Cohort Study.

**Methods:**

This cross-sectional study was conducted on 10,663 individuals aged 40–70 years old, using the baseline data of Kharamah cohort study, which started in 2014. Among all participants, 2251 individuals had a history of kidney stone. The participants’ demographic characteristics, behavioral habits, and the history of underlying diseases were investigated. The crude and Age Standardized Prevalence Rate of kidney stones was calculated. Also, logistic regression was used to identify the predictors of kidney stone. To check the goodness of fit index of the model, we used the Hosmer–Lemeshow test. All analyses were performed in STATA software.

**Results:**

The prevalence of kidney stone was estimated 21.11%. Also, the Age Standardized Prevalence Rate in men and women was calculated 24.3% and 18.7%, respectively. The mean age of the participants was 52.15 years. Higher prevalence of kidney stone was seen in women aged 40–50 years (40.47%, *p* = 0.0001) and moderate level of social economic status (31.47%, *p* = 0.03), men with overweight (44.69%, *p* < 0.0001) and those in a very high level of social economic status (35.75%, *p* = 0.001). The results of multiple logistic regression showed that the chance of having kidney stone was 1.17 times higher in diabetic individuals, 1.43 times higher in hypertensive individuals, 2.21 times higher in individuals with fatty liver, and 1.35 times higher in individuals with overweight. The level of socio economic status, male sex, and age were the other factors related to kidney stone.

**Conclusion:**

In this study, underlying diseases such as fatty liver, diabetes, and hypertension as well as age, male sex, overweight, and high social economic status were identified as important risk factors for kidney stone. Therefore, identifying individuals at risk of kidney stone and providing the necessary training can greatly help to reduce this disease. However, health policymakers should prepare preventive strategies to reduce the occurrence of kidney stone.

## Background

Kidney stone is a major cause of morbidity and affects approximately 1–15% of the world's population [1]. The types of kidney stone include calcium oxalate, calcium phosphate, uric acid, struvite, and mixed stones, among which calcium stones are the most common and include about 70 to 80% of the stones [2].

There are many differences in the prevalence rate of kidney stone worldwide, so this rate is reported as 1–5% in Asia, 5–9% in Europe, and 7–15% in North America [3]. While in Saudi Arabia, nearly 20% of individuals suffer from kidney stone, it is seen in only 4% of the population of China [4]. Iran is one of the countries which is located in the kidney stone belt [5]. According to the results of a study, the prevalence of kidney stone in Iran is reported 4.2 per thousand [6], and the highest prevalence in Iran is reported in western and southwestern provinces such as Ilam, et al. [2]. Global data show that the prevalence of kidney stone has been increasing among both sexes in the last quarter of the twentieth century [1], which may be due to environmental factors such as diet and lifestyle [7]. However, the development of diagnostic procedures for asymptomatic stones may partially explain this trend [8].

Kidney stone increases with age and is more common among men than women [9]. Also, the risk of kidney stone decreases with increasing consumption of fluids, fruits, and vegetables. Sodium restriction reduces the risk of kidney stone [7].

The recurrence rate of kidney stone is high, so at least one recurrence of kidney stone is seen during ten years in half of the individuals and in 90% of individuals over for 30 years [10]. Kidney stone increases the risk of developing chronic kidney diseases [11], end-stage renal disease [12], cardiovascular disease [13], diabetes, hypertension [14], and decreases the quality of life [15]. The burden of kidney stone is very high in both individual and community levels [16]. The annual cost of kidney stone is estimated at $ 2.1 billion in the United States in 2000, which is anticipated to increase to more than $ 4 billion by 2030 [17].

Due to the increasing trend and high recurrence of kidney stone, also imposing a heavy financial and medical burden on individuals and society [18], obtaining accurate and updated information from different regions of the world can be of great help to the decisions of health policymakers, to prepare preventive and up-to-date strategies to reduce this disease. Therefore, this study aimed to assess the prevalence and risk factors of kidney stone in the adult population of southern Iran based on the data of the Kharameh Cohort Study.

## Methods

### The study design and population

This cross-sectional study was performed using the baseline data of the Kharameh cohort study, which is a part of the large Prospective Epidemiological Studies in Iran (PERSIAN). This cohort was started in 2014 in 18 regions of Iran with the aim of investigating the factors related to non-communicable diseases. Kharameh with a population of 61,580 people is located in the south of Iran in Fars province. All people aged 40–70 years old living in this city were invited to participate in the Kharameh cohort study, and finally, 10,663 people (97.3% participation rate) were enrolled in the study. Informed consent was taken from all individuals who wished to participate in the study. The inclusion criteria were age between 40 and 70 years. The reason for selecting this group of people was that they are usually in the active period of their life and can participate in the study. In addition, the events under investigation can more probably be seen in these people. Another inclusion criterion was living in Kharameh for at least nine months so they were somewhat adapted to the cultural and environmental conditions. Exclusion criteria also included mental disorders such as mental retardation and any illness in the acute phase that is not treated. Another exclusion criterion was the unwillingness to participate in the study.

### Data collection

Data on demographic characteristics, history of diseases, and behavioral habits of the participants in the study were collected by trained interviewers and physicians during face-to-face interviews and then recorded in the questionnaires that had previously been validated in the PERSIAN Cohort Study. To check the accuracy of people's self-report in relation to the history of their disease, all their medical records were evaluated by a doctor, and questions were asked about the signs, symptoms, and treatments of their disease; if needed, the subjects were referred to other specialists for diagnostic processes.

The used information in this study included demographic characteristics of individuals (age, sex, occupation, level of education, Socio Economic Status (SES), marital status, Body Mass Index (BMI), place of residence, history of diseases such as diabetes, hypertension, fatty liver, and kidney stone, and behavioral habits of individuals including smoking, consumption of alcohol, opium, hookah, and physical activity. Questions were also asked about their use of purified water and healthy water.

SES index was calculated using variables related to their assets and by the method of Principal Component Analysis (PCA). Also, the standard questionnaire of physical activity of PERSIAN Cohort Studies was used to calculate the individuals' physical activity levels. After collecting all the information, the Metabolic Equivalent of Task (MET) index was calculated to convert physical activity into the amount of energy consumed. This index indicates the ratio of the amount of metabolism of a person's physical activity to the amount of metabolism of a person while sitting and resting [19]. Therefore, one MET equals to one kilocalorie of energy consumed per kilogram of body weight at rest. Weight was measured with minimal clothing and no shoes using a SECA scale (made in Germany), and height was measured using a standard measuring tape. BMI was also calculated by dividing the body weight (kilogram) by the square of height (m^2^). Accordingly, the participants were divided into four categories: underweight (BMI =  < 18.5), normal (18.5–24.9), overweight (25–29.9), and obese (BMI > 30).

### Kidney stone

The history of kidney stone disease was recorded and confirmed by the self-declaration of the individuals. In addition, we asked the people accompanying the participants (especially the elderly and illiterate people) about the history of suffering from kidney stone to ensure the accuracy of the answers. Also, all the medical documents (such as ultrasound, photography, documents related to surgery) were checked and confirmed by the physicians in the cohort team.

### Statistical Analysis

Frequency and percentage to describe qualitative variables and mean and standard deviation to describe quantitative variables were used. The Chi-square test or Fisher exact test was also used to examine the distribution of qualitative variables between individuals with and without kidney stone. The prevalence of actual kidney stones was calculated. Finally, logistic regression was used to evaluate the relationship between kidney stone and the factors related to it and to calculate the odds ratio. First, all the variables were examined separately in the simple logistic regression, and then variables with* p* value less than 0.2 entered the multiple logistic regression for controlling of the confounder variables and to calculate the adjusted odds ratio. Finally, after performing multiple logistic regression, to check the goodness of fit index of the model, we used the Hosmer–Lemeshow test. We performed a ROC curve analysis to examine how kidney stone is identified with other variables under study. According to the method recommended by Swets, the model's accuracy is low when the Area Under the Rock curve (AUROC) is between 0.5 and 0.7, moderately if AUROC is between 0.7 and 0.9, and high if AUROC is between 0.9 and 1 [20]. All analyses were performed in STATA software version 12 with a significance level of 95% and p-value less than 0.05.

## Results

This study was performed on 10,663 individuals aged 40–70 years who lived in Kharameh. The mean age of the participants in the study was 52.15 ± 8.22 years, and the majority of them were female (55.74%), in the age group of 40–50 years (44.72%), and did not drink purified water (79.59%) (Table 1).

**Table1:** Distribution of demographic characteristics, history of diseases and behavioral habits in individuals with and without kidney stones in the population of 40–70 years old in Kharameh cohort study

			Kidney stone in male			Kidney stone in female		
Variable	Class	Total	No (%) 3580 (75.86)	Yes (%) 1139 (24.14)	*p* value	No (%) 4832 (81.29)	Yes (%) 1112 (18.71)	*p* value
Age	40–50	4768 (44.72)	1553 (43.38)	462 (40.56)	0.24	2303 (47.66)	450 (40.47)	0.0001
	50–60	3748 (35.15)	1353 (37.79)	455 (39.95)		1545 (31.97)	395 (35.52)	
	60–70	2147 (20.14)	674 (18.83)	222 (19.49)		984 (20.26)	267 (24.01)	
Job	Unemployed	5147 (48.27)	532 (14.86)	174 (15.28)	0.19	3586 (74.21)	855 (76.89)	0.64
	Employed	5516 (51.73)	3048 (85.14)	965 (84.72)		1246 (25.79)	257 (23.11)	
Married status	Single	216 (2.03)	26 (0.73)	3 (0.26)	0.05*	151 (3.13)	36 (3.24)	0.19*
	Married	9492 (89.02)	3527 (98.52)	1132 (99.39)		3937 (81.47)	896 (80.57)	
	Widow	59 (0.55)	8 (0.22)	3 (0.26)		44 (0.91)	4 (0.36)	
	Divorced	8968.4)	19 (0.53)	1 (0.09)		7 (14.49)	176 (15.83)	
Socioeconomic status	Low	2667 (25.01)	779 (21.76)	187 (16.42)	0.0001	1410 (29.18)	291 (26.17)	0.03
	Moderate	2977 (25)	769 (21.48)	263 (23.09)		1595 (33.01)	350 (31.47)	
	High	2539 (24.98)	752 (21.01)	240 (21.07)		123 (25.58)	311 (27.97)	
	Very high	2480 (24.97)	1280 (35.75)	449 (39.42)		591 (12.28)	160 (14.39)	
Eeducation	Illiterate	5587 (52.4)	1393 (38.91)	439 (38.54)	0.66	3024 (62.58)	731 (65.74)	0.18
	Primary school	2676 (25.1)	953 (26.62)	297 (26.08)		1165 (24.11)	261 (23.47)	
	Secondary school	1136 (10.65)	566 (15.81)	169 (14.84)		336 (6.95)	65 (5.85)	
	Diploma	831 (7.62)	433 (12.09)	1449 (13.08)		197 (4.08)	34 (3.06)	
	University	451 (4.23)	235 (6.56)	85 (7.46)		110 (2.28)	21 (1.89)	
Cigarette smoking	No	7960 (74.65)	1621 (45.28)	598 (52.5)	0.0001	4681 (96.88)	1060 (95.32)	0.01
	Yes	2703 (25.35)	1959 (54.72)	541 (47.5)		151 (3.13)	52 (4.68)	
Alcohol consumption	No	10,095 (94.67)	3139 (87.68)	2130 (94.62)	0.56	4829 (99.94)	1109 (99.74)	0.08*
	Yes	568 (5.33)	441 (12.32)	121 (5.38)		3 (0.06)	3 (0.27)	
Hookah smoking	No	10,115 (994.86)	3218 (89.89)	1036 (90.96)	0.29	4767 (98.65)	1094 (98.38)	0.48
	Yes	548 (5.14)	362 (10.11)	103 (9.04)		65 (81.29)	18 (1.62)	
Opium consumption	No	8857 (83.06)	2206 (61.62)	780 (68.48)	0.0001	47,754 (98.8)	1097 (97.64)	0.68
	Yes	1806 (16.94)	1374 (38.38)	359 (31.52)		58 (1.2)	15 (1.34)	
Physical activity	Low	2670 (35.04)	1054 (29.44)	351 (30.82)	0.54	1018 (21.07)	247 (22.27)	0.55
	Moderate	2666 (25)	594 (15.59)	180 (15.8)		1531 (31.68)	361 (32.46)	
	High	2664 (24.98)	512 (14.3)	175 (15.36)		1627 (33.67)	350 (31.47)	
	Sever	2663 (24.97)	1420 (39.66)	433 (38.02)		656 (13.58)	154 (13.85)	
Drinking of purified	No	8487 (79.59)	2760 (77.09)	884 (77.61)	0.71	3932 (81.37)	911 (81.92)	067
	Yes	2176 (20.14)	820 (22.92)	255 (22.39)		900 (18.63)	201 (18.08)	
Hypertension	No	8153 (76.46)	3152 (88.04)	895 (78.58)	0.0001	3416 (70.70)	690 (62.05)	0.0001
	Yes	2510 ( (23.54)	428 (11.96)	244 (21.42)		1416 (29.3)	422 (37.95)	
Diabetes mellitus	No	9070 (85.06)	3267 (91.26)	991 (87.01)	0.0001	3966 (82.08)	846 (76.08)	0.001
	Yes	1593 (14.97)	313 (8.74)	148 (12.99)		866 (17.92)	266 (23.92)	
Fatty liver	No	9378 (87.95)	3395 (94.83)	1004 (88.15)	0.0001	4176 (86.424)	803 (72.21)	0.0001
	Yes	1285 (12.05)	1852 (5.97)	135 (11.85)		656 (13.58)	309 (27.79)	
Location	City	4416 (41.41)	1555 (43.44)	499 (43.81)	0.82	1923 (39.8)	439 (39.48)	0.84
	Village	6247 (58.59)	2025 (56.56)	640 (56.19)		2909 (60.20)	673 (60.52)	
BMI	Under weight	411 (3.85)	255 (7.12)	56 (4.92)	0.0001	86 (1.78)	14 (1.26)	0.02
	Normal	3882 (36.41)	1799 (50.25)	478 (41.97)		1312 (27.15)	293 (26.35)	
	Over weight	4451 (41.47)	1259 (35.17)	509 (44.69)		2190 (45.32)	493 (44.33)	
	Obese	919 (18)	267 (7.46)	96 (8.43)		1244 (25.75)	312 (28.06)	

*Fisher exact test

Among the participants, 2251 had a history of kidney stone. Thus, the crude prevalence and Age-standardized prevalence rate (ASPR) of kidney stone was estimated 21.11%. Also, the ASPR was estimated 24.13% (95% CI 23.7–24.6) in men and 18.7% (95% CI 18.5–18.9) in women (Table 2).

**Table 2:** Crude and age-standardized prevalence rate of kidney stone according to sex in the population of 40–70 years old in Kharameh cohort study

	95% CI			95% CI		
	Crude	Lower	Upper	ASPR	Lower	Upper
Both sexes	21.11	20.33	21.89	21.11	20.8	21.4
Male	24.13	22.9	25.38	24.13	23.7	24.6
Female	18.7	17.72	19.72	18.7	18.5	18.9

Based on Table 1, the prevalence of kidney stone is higher in women aged 40–50 years (40.47%, *p* = 0.0001), and moderate level of SES (31.47%, *p* = 0.03). Also, men with a very high level of SES (35.75%, *p* = 0.001), and overweight (44.69%, *p* < 0.0001) had a higher prevalence of kidney stone compared to other men. In addition, there was a statistically significant difference between the prevalence of kidney stone in men and women with a history of diabetes, hypertension, fatty liver, and smoking (*p* < 0.0001).

The results of simple logistic regression showed that there was a statistically significant relationship between nine variables with kidney stones. These variables included age, sex, SES, physical activity, BMI (overweight and obese), fatty liver, diabetes, hypertension, and smoking (Table 2).

In multiple logistic regression, the chance of having kidney stone in individuals aged 50–60 years was 1.12 times higher than those aged 40–50 years(OR_adj_:1.12, 95% CI 1.007–1.25). The chance of having kidney stone in men was 1.66 times higher than women (OR_adj_:1.66, 95% CI 1.49–1.85). Individuals with high level of SES had 1.19 times higher levels (OR_adj_:1.19, 95% CI 1.03–1.37), and those with very high level of SES showed 1.2 (OR_adj_:1.2, 95% CI 1.04–1.39) times higher chance of kidney stone than individuals with low levels of SES. The subjects with fatty liver (OR_adj_:2.21, 95% CI 1.93–2.52), diabetes (OR_adj_:1.17, 95% CI 1.02–1.3), and hypertension (OR_adj_:1.43, 95% CI 1.27–1.61) had 2.21, 1.17, and 1.43 times more chance of kidney stone than healthy individuals. Also, the participants with overweight had 1.35 times higher chance of kidney stone than those with underweight, after controlling the confounders (OR_adj_:1.35, 95% CI 1.03–1.78) (Table 3).

**Table 3:** Predictor variable for kidney stone based on the result of simple and multiple logistic regression in the population of 40–70 years old in Kharameh cohort study

Variable	Class	Simple logistic regression		Multiple logistic regression	
		OR (95%CI)	*p* value	OR_adj_ (95%CI)	*p* value
Age	40–50	1		1	
	50–60	1.24 (1.11–1.37)	0.0001	1.12 (1.007–1.25)	0.03
	60–70	1.24 (1.1–1.4)	0.0001	1.1 (0.96–1.26)	0.142
sex	Female	1		1	
	Male	1.38 (1.25–1.51)	0.0001	1.66 (1.49–1.85)	0.0001
Socioeconomic status	Low	1		1	
	Moderate	1.18 (1.03–1.35)	0.011	1.14 (0.99–1.)3	0.05
	High	1.26 (1.1–1.45)	0.0001	1.19 (1.03–1.37)	0.013
	Very high	1.49 (1.3–1.7)	0.0001	1.2 (1.04–1.39)	0.009
Hypertension	No	1		1	
	Yes	1.49 (1.34–1.66)	0.0001	1.43 (1.27-.161)	0.0001
Diabetes mellitus	No	1		1	
	Yes	1.38 (1.22–1.56)	0.0001	1.17 (1.02–1.3)	0.019
Fatty liver	No	1		1	
	Yes	2.2 (1.98-.2)	0.0001	2.21 (1.93–2.52)	0.0001
BMI	Under weight	1		1	
	Normal	1.2 (0.9–1.5)	0.17	1.19 (0.91–1.57)	0.19
	Over weight	1.41 (1.08–1.9)	0.011	1.35 (1.03–1.78)	0029
	Obese	1.31 (0.99–1.7)	0.055	1.28 (0.95–1.71)	0.09

**Fig. 1:**
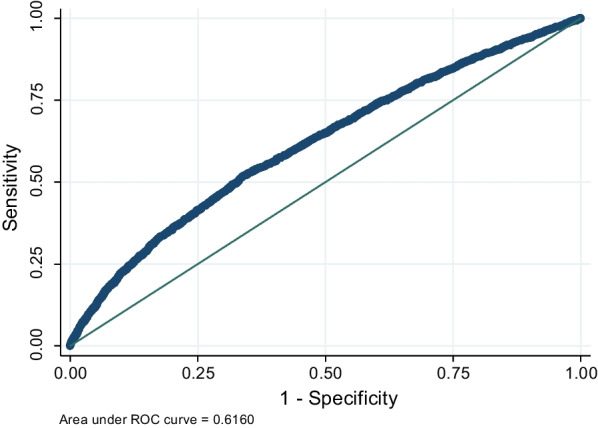
ROC curve of the related factor

The Hosmer–Lemeshow test was used to evaluate the goodness-of-fit for the adjusted model, and the results of this test showed that the model fitted well (*p* = 0.28). ROC curve analysis was performed after adjustment of the model to understand how kidney stone can be detected by the studied variables. The AUROC was estimated to be 0.615 for seven variables: age, sex, diabetes, hypertension, fatty liver, BMI, and SES (Fig. 1).

## Discussion

Kidney stone is one of the most common kidney diseases, and its prevalence is increasing. The present study was conducted on 10,663 individuals to investigate the prevalence and risk factors of kidney stone. The results of this study showed middle age, male gender, overweight, higher SES levels, diabetes, hypertension, and fatty liver as the risk factors for kidney stone.

In this study, the prevalence of kidney stone was estimated to be 21.1%, and the ASPR was higher in men than women. The estimated prevalence in the region under our study was higher than the reported prevalence in some other areas, including the United States 5.2% [21], and China 7.5% [22]. It was also reported higher than other studies in the United States and China (8.8% and 6.4%), respectively [23]. However, it was almost similar to the reported prevalence (19.1%) in the Saudi Arabia [24]. Scales et al. [9] similar to our study, reported a higher prevalence in men than in women. Also, Bihl et al. [25] stated that the prevalence of kidney stone was higher in men than in women. However, there are many reasons for the differences in the reported prevalence rates in different regions, including the method of conducting epidemiological studies, diagnostic criteria of the disease, definition of kidney stone disease, and disproportionate age and sex groups under study [23]. We should also pay attention to the differences in the climate of different regions because, in tropical regions, the body becomes dehydrated that this increases the risk of kidney stone.

Based on the results of the present study, men had a higher chance of having kidney stone, which was in line with the results of many other studies [9, 23, 26–29]. In a study in Japan, the chance of having kidney stone was reported to be 2.5 times higher in men than in women [30]. However, Scales et al. in the United States [9], contrary to our results, reported the chances of having kidney stone was higher in women than in men. We must pay attention to the role of sex hormones in the development of kidney stone. Estrogen and androgen are among the factors influencing the formation of oxalate and calcium urinary stones in men. However, estrogen prevents the formation of kidney stone in women [31]. Also, men are prone to calcium oxalate stones due to the presence of testosterone hormone [32]. Therefore, it is necessary to advise individuals to have a proper diet and enough physical activity to prevent the formation of kidney stone [31].

Based on the results of this study, the individuals in age groups of 50–60 years had a higher chance of kidney stone than those aged 40–50 years, while there was no statistically significant relationship between the prevalence of kidney stone with 60–70 year old individuals. Romer et al. in the study of the global report on the prevalence and incidence of kidney stone, stated that in the countries of Iceland, Iran, Italy, Greece, Turkey, and Germany, with increasing age, the risk of developing kidney stone also increased, but in Italy, a decreasing slope was seen in the age of over 70 years in the residents of Milan [1]. Also, Stamatelou et al. showed in their study in America that the prevalence of kidney stone decreased in women over 59 years old and in men over 69 years [21]. However, the reason for the increase in the prevalence of kidney stone in middle-aged individuals can be their laborious jobs compared to others [33], as well as less fluid consumption, improper diet, and their work stress [34]. We should note that the formation of kidney stone is related to underlying diseases such as diabetes, hypertension, and other dietary factors such as high intake of protein, sodium, and sucrose in the elderly that can lead to calcium excretion. For this reason, these individuals are more susceptible to urinary tract infections as well as the formation of kidney stone [22]. The reason for the decrease in the prevalence of kidney stone in older individuals can be the sampling methods or because elderly individuals died due to other causes, before entering the study; that is why their prevalence is underestimated [1].

Based on the results of the present study, individuals with overweight had a higher chance of having kidney stone, while there was no relationship between obesity and kidney stone. Of course, many studies have seen a direct relationship between the increase in BMI and the formation of kidney stone [9, 35–38]. However, Shahidi et al. [39] in Iran did not find a statistically significant relationship in this regard. Eisener et al. [40] revealed that an increase in BMI was associated with several risk factors for urinary tract diseases, including an increase in urinary sodium and a decrease in pH in men, as well as an increase in uric acid, urinary sodium, and a decrease in urinary citrate in women. Physiologically, obesity is related to increased excretion of calcium and uric acid, as well as increased urinary acidity, all of which contribute to increased risk of kidney stone formation [9].

In this study, increasing levels of SES showed a statistically significant relationship with the risk of kidney stone formation. Also, this relationship was seen in the northern regions of Taiwan and China, which have a high level of SES [41]. In Japan, India and Iran, the distribution of kidney stone was also reported at higher levels of SES [28, 42], but Musluanglo et al. [43] in their study did not observe a significant relationship between the level of SES and the occurrence of kidney stone. Anyway, this chronic metabolic disease can be seen due to high calorie intake in populations with higher living standards because these individuals usually have an unhealthy lifestyle and eating habits that can affect the formation of kidney stone [43]. In addition, we must keep in mind that kidney stone in individuals who live in low-level areas may remain undiagnosed due to lack of access to diagnostic and treatment services [44].

Diabetic individuals in our study had a higher chance of having kidney stone; the results of many other studies were similar to ours [9, 23, 28, 39, 45, 46]. Weinberg et al. [47] also stated that the severity of type 2 diabetes was an important risk factor for developing kidney stone. But, unlike the results of our study, Kabey et al. did not report a positive relationship [48]. However, the relationship between diabetes and kidney stone is explained in the way that insulin receptors are expressed in the epithelium of the renal tubules, and insulin participates in the ammonia removal of the renal tubules. In the diabetic state, when insulin resistance occurs, there is a disturbance in the ammonia removal of the kidney tubules. As a result, acidic urine and urinary stones are produced [47, 49].

In the present study, hypertension was found to be another risk factor for the formation of kidney stone. This relationship was also seen in Shahidi's study [39]. Taylor and his colleagues in their study did not find a direct relationship in this regard [38]. Anyway, metabolic acidosis and hypocitraturia in hypertensive individuals play a key role in the formation of kidney stone. However, we should consider the role of other confounding factors [39].

Fatty liver was also recognized as another important factor causing kidney stone in our study. In line with our study, many other studies have identified fatty liver as an important risk factor for kidney [50–53]. In a study in Israel, the risk of kidney stone formation in individuals with fatty liver was reported to be 3.2 times [54]. However, in a study in Iran, fatty liver was introduced as a risk factor for kidney stone in men and a protective factor in women [51]. Yet, the biological mechanism of how fatty liver increases the incidence of kidney stone is unknown. In general, the low antioxidant level in these individuals increases their susceptibility to kidney stone. Oxidative metabolisms, which are related to the formation of urinary stones, due to the abundance of unsaturated fatty acids, are combined with long chains of lipids and lead to lipid peroxidation, which may affect the risk of calcium oxalate stones formation [14]. We should not neglect the presence of other common risk factors for kidney stone such as obesity and diabetes in individuals with fatty liver.

In our study, there was no statistically significant relationship between the use of purified water and kidney stone. Pubali et al. [55] also did not show any statistically significant relationship. Anyway, in this regard, we should consider the role of water hardness in different geographical areas as well as weather conditions. Basiri et al. [56] in their study in different cities of Iran showed that the hardness of tap water had no significant relationship with the regional prevalence of urinary tract infections.

In the present study, similar to some other studies, there was no statistical relationship between smoking and kidney stone [57–59]. However, Khalili et al. [28] in their study introduced smoking as a protective factor against kidney stone, some other studies have identified smoking as an independent risk factor for developing kidney stone [60–62]. However, smoking, by increasing antidiuretic plasma hormone, leads to a decrease in the urinary volume and an increase in the production of crystal stones. Also, smoking causes kidney damage by increasing oxidative stress. The level of cadmium and lead increases in smokers, which leads to the formation of kidney stone. Anyway, the reason for the difference in the results obtained in different studies can be the different age groups in the studies, sample size of the studies, as well as cultural and traditional factors governing the societies, such as smoking [28, 61, 63].

In our study, similar to some other studies, there was no statistically significant relationship between physical activity and kidney stone [3, 61, 64], while some other studies showed physical activity as a protective factor for kidney stone [57, 65]. About 60% of the people in our study had a low and moderate level of physical activity, which is almost a high percentage. Anyway, due to the cultural context of the region under investigation and the lack of sufficient sports facilities in this region, the level of physical activity is relatively low. For a detailed understanding of the mechanism and effect of physical activity on kidney stone, it is recommended that more extensive studies should be conducted with an emphasis on physical activity.

Finally, ROC analyses showed that variables of sex, BMI, age, history of hypertension, fatty liver, diabetes, and SES were less accurate for the prediction of kidney stone.

### Limitations

Although our results are derived from a cohort study with a large sample size that examined many variables, due to the cross-sectional nature of our study, we are not able to accurately estimate causal relationships because the time sequence is not clear. In addition, given that the condition of having kidney stone has been reported by the participants themselves, we may be faced with recall bias and our estimates are less than the reality reported.

## Conclusion

The results of the present study showed that, middle age, male gender, high SES level, diabetes, hypertension, and fatty liver are important risk factors for kidney stones in adults aged 40–70 years. Considering the high prevalence of kidney stone in the population under study, preventive measures should be taken to reduce the incidence of kidney stones, especially by considering modifiable risk factors such as diabetes and high hypertension Also, by providing the necessary education about risk factors to people aged 40–70, it is possible to help reduce the incidence and burden of kidney stones. The results of the present study help health policy makers to develop preventive guidelines.

## Data Availability

The datasets used and/or analysed during the current study available from the corresponding author on reasonable request.
